# Evaluation of Impact of Factors Affecting CT Radiation Dose for Optimizing Patient Dose Levels

**DOI:** 10.3390/diagnostics10100787

**Published:** 2020-10-05

**Authors:** Ching-Ching Yang

**Affiliations:** 1 Department of Medical Imaging and Radiological Sciences, Kaohsiung Medical University, Kaohsiung 80708, Taiwan; cyang@kmu.edu.tw; 2 Department of Medical Research, Kaohsiung Medical University Chung-Ho Memorial Hospital, Kaohsiung 80708, Taiwan

**Keywords:** CT dose metric, diagnostic reference level, protocol optimization

## Abstract

The dose metrics and factors influencing radiation exposure for patients undergoing head, chest, and abdominal computed tomography (CT) scans were investigated for optimization of patient dose levels. The local diagnostic reference levels (DRLs) of adult CT scans performed in our hospital were established based on 28,147 consecutive examinations, including 5510 head scans, 9091 chest scans, and 13,526 abdominal scans. Among the six CT scanners used in our hospital, four of them are 64-slice multi-detector CT units (MDCT_64_), and the other two have detector slices higher than 64 (MDCT_H_). Multivariate analysis was conducted to evaluate the effects of body size, kVp, mAs, and pitch on volume CT dose index (CTDI_vol_). The local DRLs expressed in terms of the 75th percentile of CTDI_vol_ for the head, chest, and abdominal scans performed on MDCT_64_ were 59.32, 9.24, and 10.64 mGy, respectively. The corresponding results for MDCT_H_ were 57.90, 7.67, and 9.86 mGy. In regard to multivariate analysis, CTDI_vol_ showed various dependence on the predictors investigated in this study. All regression relationships have coefficient of determination (R^2^) larger than 0.75, indicating a good fit to the data. Overall, the research results obtained through our workflow could facilitate the modification of CT imaging procedures once the local DRLs are unusually high compared to the national DRLs.

## 1. Introduction

Computed tomography (CT) is one of the most important radiologic modalities performed in modern medical practice. Since its introduction over 45 years ago, CT scanner has evolved from single-slice CT (SSCT) to multi-detector CT (MDCT). Currently, the major CT manufacturers offer 64-, 128-, 256-, and 320-slice MDCT systems. Technology advancements and innovations have extended the clinical applications of CT imaging. However, the radiation dose from CT examinations has raised serious concerns on patient safety and public health. Studies have reported that CT contributes 49–68% of the collective dose from diagnostic radiologic examinations [1,2,3]. Several dose reduction strategies have been proposed to keep patient doses as low as reasonably achievable (ALARA) in the past decades [2,4,5]. Implementation of diagnostic reference levels (DRLs) has been demonstrated to be a powerful tool to identify unusually high patient doses from radiologic examinations [6,7]. The concept of DRLs was initially introduced by the International Commission on Radiological Protection (ICRP) in 1991 [8]. The DRLs of CT examinations are generally expressed in terms of volume CT dose index (CTDI_vol_) or dose-length product (DLP). National DRLs for CT examinations, which are commonly set at the third quartile of the dose distribution from a national survey, have been reported in several countries [9,10,11,12,13,14,15,16,17,18,19]. DRLs can also be established based on the amount of ionizing radiation at a local healthcare facility, which are referred to as local DRLs. The CT image quality sufficient for clinical needs may vary among different institutions, so local DRLs should be reviewed to allow better management and optimization of patient radiation exposure [7]. Therefore, in the first part of this study, local DRLs were established based on consecutive CT examinations performed in our hospital for adult patients undergoing head, chest, and abdominal scans. Local DRLs could identify unusually high doses being delivered, but they provide no information on how to tailor the scan parameters to reduce radiation dose. To enable better management of patient radiation exposure, the impact of factors influencing CT dose metrics was investigated through multivariate analysis in the second part of this study.

## 2. Materials and Methods

### 2.1. CT Examinations

Consecutive CT examinations for adult patients (age ≥ 20 years) undergoing head, chest, and abdominal scans in our hospital between November 2017 and October 2018 were investigated in this study. CT examinations were included whether they were performed with or without intravenous contrast. Approval was gained from the institutional Human Research Ethics Committee to access these practice data (approval code: CGH-P109025; approved on April 23, 2020). CT examinations were performed on 6 scanners in 4 different models, including 3 Somatom Definition AS, 1 Somatom Definition Flash (Siemens Healthcare, Erlangen, Germany), 1 Aquilion, and 1 Aquilion One (Canon Medical Systems, Otawara, Japan). Flash is a dual-source scanner, and the other 3 models are single-source scanner. AS, Flash, Aquilion, and Aquilion One have 32, 64, 64, and 320 physical detector rows, respectively, which result in axial coverage of 19.2, 38.4, 32, and 160 mm. AS, Flash, and Aquilion One are equipped with double sampling techniques (z-flying focal spot (Siemens Healthcare, Erlangen, Germany) for AS and Flash; ConeXact double slice reconstruction (Canon Medical Systems, Otawara, Japan) for Aquilion One). Thus, the corresponding detector slices of the 4 models are 64, 128, 64, and 640. Among these CT scanners used in our hospital, 4 of them are 64-slice units (MDCT_64_: 3 AS + 1 Aquilion), and the other two have detector slices higher than 64 (MDCT_H_: 1 Flash + 1 Aquilion One). 

### 2.2. Patient Characteristics, Scan Parameters, and Dose Metrics

Scan data retrieved through Picture Archiving and Communication System (PACS) connection were deidentified and uploaded to a server installed Radimetrics (Bayer HealthCare, Whippany, NJ, USA) to generate a record listing patient characteristics, scan parameters, and dose metrics. The patient characteristics used in this study include gender, age, and body size. Patient gender and age were extracted from Digital Imaging and Communications in Medicine (DICOM) tag. Automatic measurement of body size was conducted by Radimetrics through calculating the average of patient’s effective diameter at each axial CT slice. The effective diameter is the diameter of a circle equal to that of the patient’s cross-section. With regards to scan parameters, the following information in DICOM tag were extracted, including scan region, protocol name, device, tube voltage, tube current, rotation time, slice thickness, collimation, pitch, scan length, and field of view (FOV). CT examinations collected in this study were categorized as CT angiography (CTA) scans or CT scans excluding CTA according to the description of the extracted protocol name. Considering dose metrics, CTDI_vol_ and DLP were extracted from either DICOM tag or dose report sheet to establish local DRLs. The CTDI_vol_ in head scans is based on the 16-cm-diameter phantom, while the CTDI_vol_ in chest and abdominal scans is based on the 32-cm-diameter phantom. In addition to CTDI_vol_ and DLP, effective dose was also calculated for establishing local DRLs, where effective dose = DLP*k. The k factors for head, chest, and abdominal CT examinations were 0.019, 0.0145, and 0.0170 mSv/mGy-cm, respectively. The k factors were calculated based on tissue-weighting factors in ICRP publication 103 [20]. The first, second, third quartiles, and the mean value of CT dose metrics (including CTDI_vol_, DLP and effective dose) were calculated associated with scan region described in DICOM tag, i.e., head, chest, and abdomen.

### 2.3. Multivariate Analysis

The automatic tube current modulation (ATCM) technique is generally used in CT scans performed in our hospital to achieve predetermined image quality for patients with different body habitus. CareDose4D (Siemens Healthcare, Erlangen, Germany) is the ATCM system for AS and Flash, while SureExposure (Canon Medical Systems, Otawara, Japan) is the ATCM system for Aqilion and Aquilion One. Although these two ATCM systems are based on different operating mechanisms [21,22], it was expected that body size should correlate with radiation exposure when ATCM was applied. The CT scanners installed in our hospital has no automatic tube voltage modulation technique, so kVp was selected by operator, mainly according to patient’s body mass index (BMI). The combination of manually selected kVp and automatically modulated mA would together contribute to the radiation exposure, but there is no straightforward relationship between the selection of scan parameters and radiation dose received by patients. Therefore, multiple linear regression methods were used to analyze the impact of a variety of factors on CTDI_vol_ for adult patients undergoing head, chest, and abdominal scans with or without contrast in our hospital. In our routine practice, MDCT_H_ is the first choice to perform chest scans because MDCT_H_ provides better temporal resolution and longer axial coverage to reduce motion artifacts. Contrarily, MDCT_64_ is the first choice to conduct head scans because head scans are less affected by motion artifacts. It has been reported that scanner design also affects the radiation dose required to achieve a particular image quality [4,5]. Since CT examinations are scheduled according to scanner capabilities to reduce motion artifacts and body part examined, data were stratified into MDCT_64_ and MDCT_H_ to investigate the potential resulting impact on radiation dose. 

Because of the use of ATCM, body size was chosen to be one of the predictors in the multivariate model. Moreover, previous studies have reported that radiation dose is proportional to kVp^2^, mA and 1/pitch [23,24,25], so they were also selected to be the predictors in the multivariate model. The model to explain the relationship between independent and dependent variables was

(1) $${\mathrm{CTDI}}_{\mathrm{vol}}{\;=\;B}_{0}{\;+\;B}_{1}*\left(\mathrm{body}\;\mathrm{size}\right){\;+\;B}_{2}{*(\mathrm{kVp}2)\;+\;B}_{3}*\left(\mathrm{mAs}\right){\;+\;B}_{4}*\left(1/\mathrm{pitch}\right)$$

where *B*_0_ to *B*_4_ are the regression coefficients (*B*) to be estimated. The body size in Equation (1) is the average of patient’s effective diameter at each axial slice. The standard regression coefficients (β) were calculated to assess the relative importance of each independent variable. Student’s t test and variance inflation factor (VIF) were used as criteria in screening the potential regression model. A predictor was considered statistically significant if |*t*| > 2. A maximal VIF value in excess of 10 was regarded as an indication that multicollinearity may be unduly influencing the least square estimates. The coefficient of determination (*R*^2^) was calculated to assess the strength of the functional regression model. The statistical analysis algorithms were implemented in MATLAB 7.1 (The Mathworks, Natick, MA, USA).

## 3. Results

### 3.1. Radiation Dose Metrics

Overall, 28,147 CT examinations were investigated in this study, including 5510 head scans (male: 2676, female: 2854), 9091 chest scans (male: 4730, female: 4361), and 13,526 abdominal scans (male: 6285, female: 7241). Table 1 summarizes the first, second, and third quartiles and the mean value of CTDI_vol_ for adult patients undergoing head, chest, and abdominal scans performed using MDCT_64_ and MDCT_H_. The corresponding results for DLP and effective dose are reported in Table 2 and Table 3, respectively. The mean values of scan length for head, chest, and abdominal scans performed on MDCT_64_ are 175.62, 408.95, and 373.85 mm, respectively. The corresponding results are 195.31, 373.26, and 347.33 mm in MDCT_H_. 

### 3.2. Multivariate Analysis for MDCT_64_

**Figure 1:**
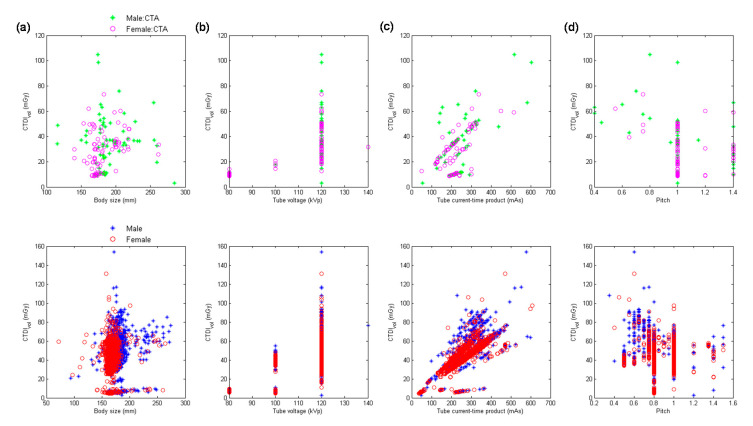
Scatter plots of CTDI_vol_ versus (**a**) body size, (**b**) kVp, (**c**) mAs, and (**d**) pitch for head CTA scans (**upper** row) and head scans excluding CT angiography (CTA) (**lower** row) performed using 64-slice multi-detector CT units (MDCT_64_ ).

Figure 1 demonstrates the scatter plots of CTDI_vol_ versus body size, kVp, mAs, and pitch for head performed using MDCT_64_. The upper row displays the distribution for head scans categorized as CTA, and the lower row displays the distribution for head scans excluding CTA. Table 4 summarizes the results of multiple linear regression analysis for head scans from MDCT_64_. It was found in Table 4 that mAs (β = 0.66) was the most significant predictor of CTDI_vol_ for head CTA, followed by kVp^2^ (β = 0.63) and 1/pitch (β = 0.42). The corresponding results for head scans excluding CTA are mAs (β = 0.90), 1/pitch (β = 0.51), and kVp^2^ (β = 0.44). Body size was not a significant predictor of CTDI_vol_ for head scans performed using MDCT_64_ (|*t*| < 2). 

**Figure 2:**
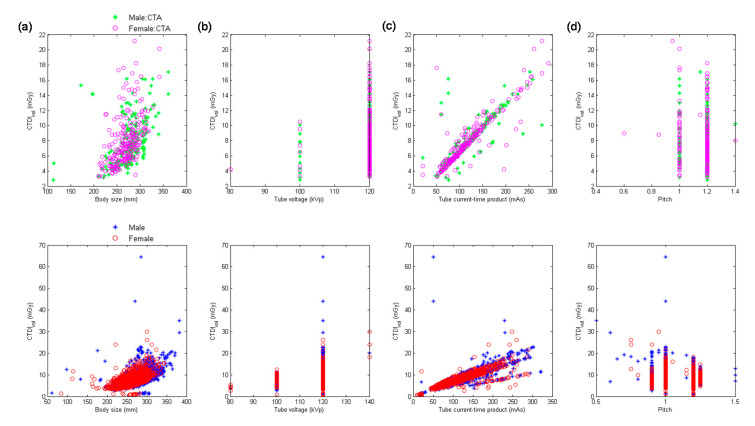
Scatter plots of CTDI_vol_ versus (**a**) body size, (**b**) kVp, (**c**) mAs, and (**d**) pitch for chest CTA scans (**upper** row) and chest scans excluding CTA (**lower** row) performed using MDCT_64_.

Figure 2 demonstrates the scatter plots of CTDI_vol_ versus body size, kVp, mAs, and pitch for chest CTA scans and chest scans excluding CTA performed using MDCT_64_. Table 5 summarizes the results of regression analysis for chest scans from MDCT_64_. It was found in Table 5 that mAs (β = 0.87) was the most significant predictor of CTDI_vol_ for chest CTA, followed by kVp^2^ (β = 0.33), 1/pitch (β = 0.29) and body size (β = 0.08). The corresponding results for chest scans excluding CTA are mAs (β = 0.82), kVp^2^ (β = 0.32), 1/pitch (β = 0.31), and body size (β = 0.07). 

**Figure 3:**
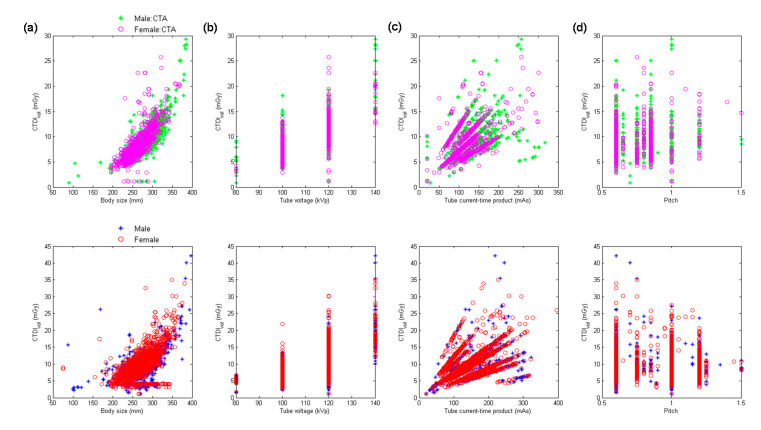
Scatter plots of CTDI_vol_ versus (**a**) body size, (**b**) kVp, (**c**) mAs, and (**d**) pitch for abdominal CTA scans (**upper** row) and abdominal scans excluding CTA (**lower** row) performed using MDCT_64_.

Figure 3 demonstrates the scatter plots of CTDI_vol_ versus body size, kVp, mAs, and pitch for abdominal CTA scans and abdominal scans excluding CTA performed using MDCT_64_. Table 6 summarizes the results of regression analysis for abdominal scans from MDCT_64_. It was found in Table 6 that kVp^2^ (β = 0.78) was the most significant predictor of CTDI_vol_ for abdominal CTA, followed by mAs (β = 0.73), 1/pitch (β = 0.43), and body size (β = 0.12). The corresponding results for abdominal scans excluding CTA are mAs (β = 0.97), kVp^2^ (β = 0.76), 1/pitch (β = 0.66), and body size (β = 0.07). 

The regression models for the head, chest, and abdominal CTA scans from MDCT_64_ yielded an *R*^2^ of 0.96, 0.82, and 0.92, respectively. The corresponding results for CT scans excluding CTA performed using MDCT_64_ are 0.91, 0.82, and 0.91. High multicollinearity was not observed among independent variables in any modelling for CT scans from MDCT_64_ (VIF < 10).

### 3.3. Multivariate Analysis for MDCT_H_

**Figure 4:**
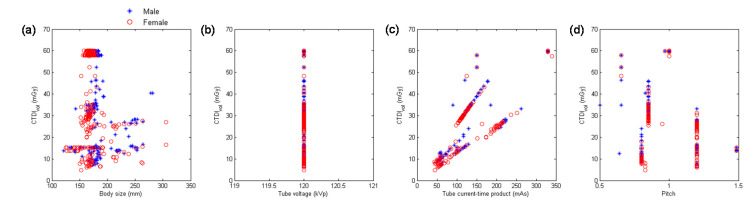
Scatter plots of CTDI_vol_ versus (**a**) body size, (**b**) kVp, (**c**) mAs, and (**d**) pitch for the head scans performed using MDCT_H_.

In our hospital, MDCT_H_ is not preferred for head CTA, so the head scans performed using MDCT_H_ were categorized as head scans excluding CTA. Figure 4 demonstrates the scatter plots of CTDI_vol_ versus body size, kVp, mAs, and pitch for the head scans performed using MDCT_H_. Table 7 summarizes the results of regression analysis for head scans from MDCT_H_. Since head scans were performed with fixed 120-kVp tube voltage (Figure 4b) on MDCT_H_, kVp^2^ was not a predictor of CTDI_vol_. As can been seen in Table 7, 1/pitch (β = 0.69) was the most significant predictor of CTDI_vol_, followed by mAs (β = 0.68), while body size was not as significant predictor of CTDI_vol_ (|*t*| < 2). 

**Figure 5:**
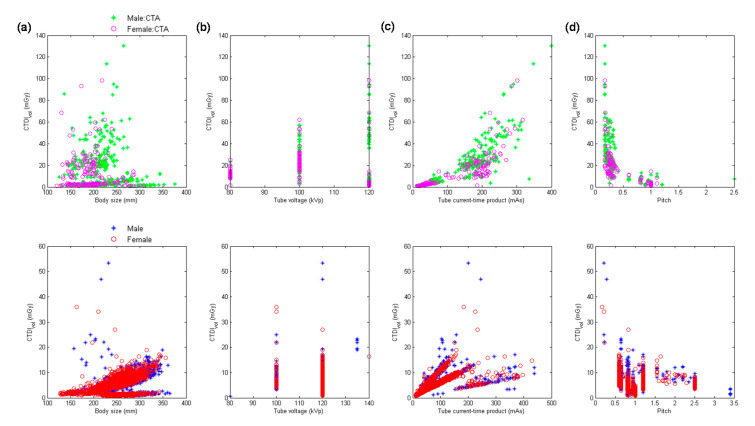
Scatter plots of CTDI_vol_ versus (**a**) body size, (**b**) kVp, (**c**) mAs, and (**d**) pitch for chest CTA scans (**upper** row) and chest scans excluding CTA (**lower** row) performed using MDCT_H_.

Figure 5 demonstrates the scatter plots of CTDI_vol_ versus body size, kVp, mAs, and pitch for chest CTA scans and chest scans excluding CTA performed using MDCT_H_. Table 8 summarizes the results of regression analysis for chest scans from MDCT_H_. It was found in Table 8 that mAs (β = 0.70) was the most significant predictor of CTDI_vol_ for chest CTA, followed by 1/pitch (β = 0.65), kVp^2^ (β = 0.62), and body size (β = 0.02). As for chest scans excluding CTA, the most significant predictor was mAs (β = 0.79), followed by 1/pitch (β = 0.62), body size (β = 0.15), and kVp^2^ (β = 0.08). 

**Figure 6:**
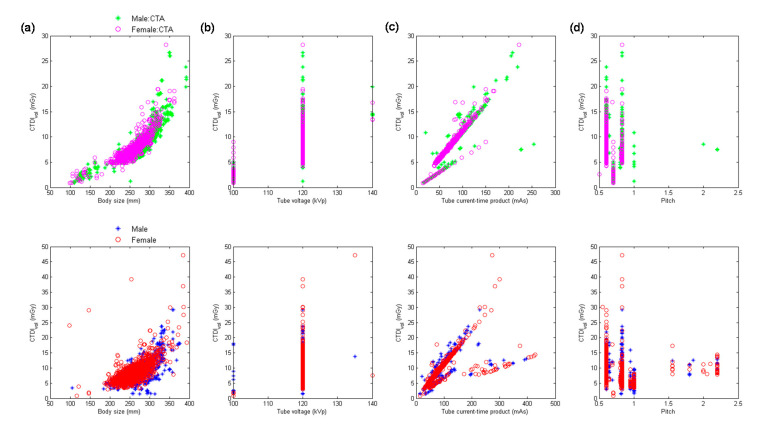
Scatter plots of CTDI_vol_ versus (**a**) body size, (**b**) kVp, (**c**) mAs, and (**d**) pitch for abdominal CTA scans (**upper** row) and abdominal scans excluding CTA (**lower** row) performed using MDCT_H_.

Figure 6 demonstrates the scatter plots of CTDI_vol_ versus body size, kVp, mAs and pitch for abdominal CTA scans and abdominal scans excluding CTA performed using MDCT_H_. Table 9 summarizes the results of regression analysis for abdominal scans from MDCT_H_. It was found in Table 9 that mAs (β = 0.78) was the most significant predictor of CTDI_vol_ for abdominal CTA, followed by body size (β = 0.18), kVp^2^ (β = 0.10), and 1/pitch (β = 0.02). The corresponding results for abdominal scans excluding CTA are mAs (β = 0.59), body size (β = 0.36), 1/pitch (β = 0.31) and kVp^2^ (β = 0.08). The regression models for the chest and abdominal CTA scans from MDCT_H_ yielded an R^2^ of 0.93 and 0.92, respectively. With regards to CT scans excluding CTA performed using MDCT_H_, the R^2^ was 0.89, 0.79, and 0.79 for head, chest, and abdominal scans, respectively. High multicollinearity was not observed among independent variables in any modelling for CT scans from MDCT_H_ (VIF < 10).

## 4. Discussion

As seen in Table 1, Table 2 and Table 3, the CT dose metrics from MDCT_H_ are generally lower than those from MDCT_64_, except the 75th percentile of DLP and effective dose in head scans. Iterative reconstruction techniques have emerged in CT as a viable alternative to the standard algorithm of filtered back-projection (FBP). Use of iterative reconstruction techniques has been demonstrated as an effective method for radiation dose savings through noise reduction in CT image processing [26,27,28]. In our hospital, iterative reconstruction is applied in some CT protocols performed on Flash and Aquilion One, which may contribute to the difference in tube current-time product between MDCT_64_ and MDCT_H_. The mean values of tube-current time product in MDCT_64_ are 285.73 mAs in males and 280.31 mAs in females for head scans, 122.35 mAs in males and 112.59 mAs in females for chest scans, and 127.89 mAs in males and 121.80 mAs in females for abdominal scans. With regards to MDCT_H_, the mean values of tube-current time product are 151.97 mAs in males and 153.01 mAs in females for head scans, 82.46 mAs in males and 76.33 mAs in females for chest scans, and 79.49 mAs in males and 76.02 mAs in females for abdominal scans. With the help of iterative reconstruction, a lower tube current-time product can be used to achieve the same noise level, thus reducing patient radiation exposure. The level of noise reduction can be adjusted for either the iterative reconstruction method implemented in Flash (Sinogram Affirmed Iterative Reconstruction, SAFIRE) or Aquilion One (Adaptive Iterative Dose Reduction, AIDR). In SAFIRE algorithm, five presets can be adjusted, where strength 1 is the weakest level of noise reduction and strength 5 is the strongest. As for AIDR algorithm, four presets can be adjusted for the level of noise reduction, including weak, mild, standard, and strong. In our routine practice, images are usually reconstructed following manufacturers’ recommendations, i.e., strength 3 for SAFIRE and standard for AIDR. Further dose reduction could be achieved by using iterative reconstruction with a stronger noise reduction strength, but it may degrade image quality due to degradation of spatial resolution and change in image texture. Using sharp kernel is a possible strategy to reduce the degradation of spatial resolution. However, the artificial look due to strong noise reduction strength could be a diagnostic challenge. Recently, deep learning-based image reconstruction methods have been proposed by several studies [29,30,31]. Promising results have been reported in preserving subtle structures at low radiation exposure. However, further investigations are needed to thoroughly understand the performance characteristics of deep learning-based image reconstruction methods. With regards to the dose metrics of head scans, the CTDI_vol_-based local DRLs for MDCT_64_ is higher than those for MDCT_H_ (Table 1), but the DLP-based local DRLs for MDCT_64_ is lower than those for MDCT_H_ (Table 2). This could be explained by the longer scan length used in MDCT_H_ when performing head scans. The mean values of scan length for the head scans performed using MDCT_64_ are 182.71 mm and 168.92 mm in male and female patients, respectively. The corresponding results are 200.75 mm and 190.54 mm in MDCT_H_. Using a longer collimation to ensure the complete region of clinical interest is included in the scan range may exposure patient outside the region of clinical interest, consequently resulting in a longer scan length. This phenomenon is less prominent in CT examinations covering longer regions of clinical interest, so it was observed only in head scans but not in chest or abdominal scans.

With regards to the multivariate analysis, different power functions, kVp^n^ (*n* ranged from 2 to 3.5), were assessed to achieve the best fit to the consecutive CT examinations. The regression model with the square of tube voltage showed the highest R^2^, so it is the model used in this study. Slice thickness is an important factor of radiation dose, but it is usually fixed for the same scan series in our routine clinical practice. For example, the smallest slice thickness is used to conduct coronary CT angiography, which is 0.6 mm in Flash and 0.5 mm in Aquilion One. Another example is coronary artery calcification scan which uses a slice thickness of 3 mm to derive Agatston score. On the other hand, the same scan series could be performed with a range of kVp, mAs or pitch. To achieve a better fit to the consecutive CT examinations, slice thickness was not used as a predictor of the regression model. All regression relationships have R^2^ larger than 0.75, indicating a good fit to the data. The lowest R^2^ was found in abdominal scans excluding CTA performed on MDCT_H_, which may be due to the anatomical variations in abdomen (Figure 6a, lower row). For adult patients, variation in chest or abdominal circumference is obvious for patients with different body habitus, so the CTDI_vol_, which depends on the manually selected kVp and automatically modulated mA, would highly correlate with body size. This phenomenon can be observed in Table 5, Table 6, Table 7, Table 8 and Table 9. However, the difference in head circumference between normal and obese patients is little, which may explain the weak correlation observed between CTDI_vol_ and body size for head scans (Figure 1a–c) and Table 4 for MDCT_64_; Figure 4a–c and Table 7 for MDCT_H_). As seen in Figure 1d and Figure 6d, there were negative correlations between CTDI_vol_ and pitch. A helical pitch greater than one implies gap between the x-ray beams, while a pitch less than one indicates overlapped beam exposure [2,4,5]. Hence, reducing pitch would increase image quality and CTDI_vol_. No substantial difference in data distribution was observed between CTA scans and CT scans excluding CTA, except for the chest scans performed on MDCT_H_ (Figure 5). This phenomenon may be due to the high image quality required in coronary CTA, which is only performed on MDCT_H_ in our hospital because wide-volume scanners can reduce misalignment artifacts and improve temporal resolution. Due to the small size and rapid motion of the coronary arteries, imaging of the coronary arteries requires high spatial resolution (small voxel size) and also high temporal resolution (short rotation time). Hence, high tube currents are used to provide a sufficient number of photons for adequate image quality. The greatest flexibility for the reconstruction of motion-free cardiovascular images is provided by retrospectively gated helical acquisitions, which use a low helical pitch (0.2–0.5) [32,33]. However, high tube currents and low pitch factor both result in high radiation exposure to the patients. To reduce radiation dose from coronary CTA, the prospectively gated transverse acquisition should be considered for patients with stable and low heart rates [32,33,34]. 

**Figure 7:**
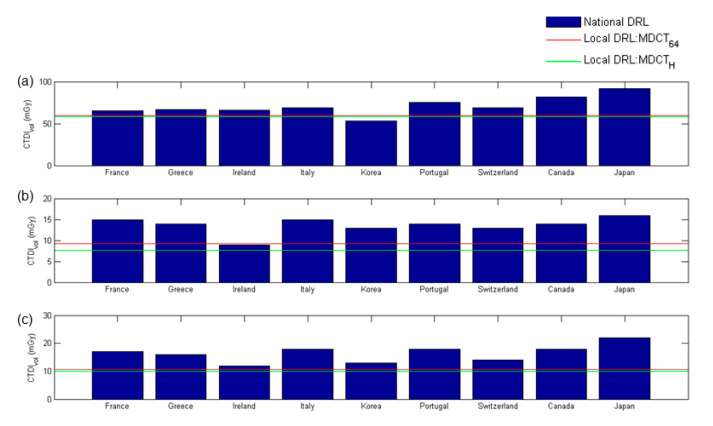
Comparison of local diagnostic reference levels (DRLs) expressed in terms of the 75th percentile CTDI_vol_ with published national DRLs for adult patients undergoing (**a**) head, (**b**) chest, and (**c**) abdominal scans.

**Figure 8:**
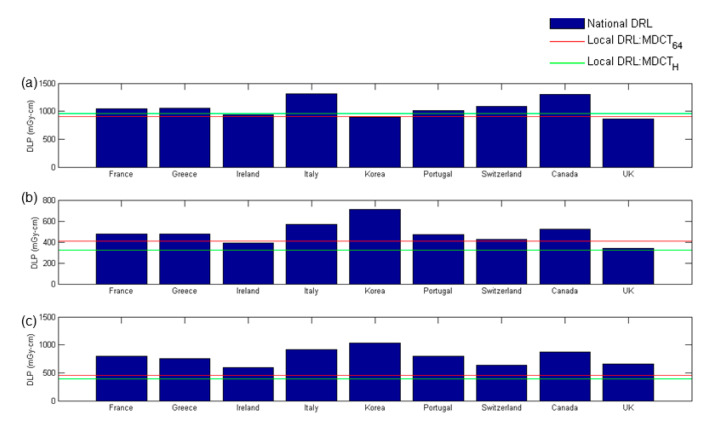
Comparison of local DRLs expressed in terms of the 75th percentile DLP with published national DRLs for adult patients undergoing (**a**) head, (**b**) chest, and (**c**) abdominal scans.

Figure 7 demonstrates a comparison of the established local DRLs expressed in terms of the 75th percentile CTDI_vol_ and the published national DRLs from national surveys in France [10], Greece [11], Ireland [12], Italy [13], Korea [14], Portugal [15], Switzerland [16], Canada [17], and Japan [18]. It was observed that the CTDI_vol_-based local DRLs established in this study are generally lower than the published national DRLs, except for the head scans in Korea and chest scans in Ireland. Figure 8 demonstrates a comparison of the established local DRLs expressed in terms of the 75th percentile DLP and the published national DRLs from national surveys in France [10], Greece [11], Ireland [12], Italy [13], Korea [14], Portugal [15], Switzerland [16], Canada [17], and the UK [19]. The DLP-based local DRLs established in this study are generally lower than the national DRLs, except for the head scans in Ireland, Korea, and the UK and chest scans in Ireland and the UK. The abdominal scans performed in our hospital only include from the top of the diaphragm to the top of the iliac crest, while the scan length of abdominal scans covers both abdomen and pelvis in some countries listed in Figure 8. In addition, patient height also affects the selection of scan length. These may explain why the DLP-based local DRLs for abdominal scans established in this study are much lower than the national DRLs compared with CTDI_vol_-based DRLs. Periodic large-scale patient dose surveys and protocol optimization are very well established in the UK and some European countries, so the radiation technologists, medical physicists, and radiologists from these countries may have greater awareness and concern regarding the potential risk of radiation. Although differences between their data and ours do exist, their views are important references when optimizing patient dose levels. According to the results in Table 4, Table 5, Table 6, Table 7, Table 8 and Table 9, protocol optimization could be conducted efficiently by modifying the significant factors in the regression models. 

## 5. Conclusions

Conducting dose surveys to establish DRLs is a useful method to understand CT radiation doses from different scanners, institutions, and countries. Moreover, information from dose surveys can also be used for optimizing patient dose levels to achieve the ALARA principle. In the first part of this study, the local DRLs for adult patients undergoing head, chest, and abdominal scans were established based on consecutive CT examinations performed in our hospital and compared with the published national DRLs. Local DRLs could identify unusually high doses being delivered, but they provide no information on how to tailor the scan parameters to reduce radiation dose. To enable better management of patient radiation exposure, the impact of factors influencing CT dose metrics was investigated through multivariate analysis in the second part of this study. Overall, the research results obtained through our workflow could facilitate the modification of CT imaging procedures once the local DRLs are unusually high compared to the national DRLs. 

## Figures and Tables

**Table 1:** Volume CT dose index (CTDI_vol_) (mGy) from consecutive adult computed tomography (CT) examinations.

	MDCT_64_			MDCT_H_		
	Head	Chest	Abdomen	Head	Chest	Abdomen
Number of Acquisition	*n* = 4984	*n* = 3670	*n* = 8243	*n* = 546	*n* = 5421	*n* = 5283
25th percentile	39.57	5.74	7.03	15.20	2.66	6.40
50th percentile	50.82	7.29	8.72	34.49	5.74	7.94
75th percentile	59.32	9.24	10.64	57.90	7.67	9.86
Mean	48.40	7.87	9.09	37.68	6.58	8.44

**Table 2:** Dose-length product (DLP) (mGy-cm) from consecutive adult CT examinations.

	MDCT_64_			MDCT_H_		
	Head	Chest	Abdomen	Head	Chest	Abdomen
Number of Acquisition	*n* = 4984	*n* = 3670	*n* = 8243	*n* = 546	*n* = 5421	*n* = 5283
25th percentile	668.00	190.00	203.00	260.40	57.00	161.30
50th percentile	807.00	261.00	325.50	711.50	200.20	248.00
75th percentile	908.00	411.40	452.98	954.00	324.25	386.95
Mean	851.84	332.64	350.14	646.39	234.88	292.29

**Table 3:** Effective dose (mSv) from consecutive adult CT examinations.

	MDCT_64_			MDCT_H_		
	Head	Chest	Abdomen	Head	Chest	Abdomen
Number of Acquisition	*n* = 4984	*n* = 3670	*n* = 8243	*n* = 546	*n* = 5421	*n* = 5283
25th percentile	1.68	4.43	3.41	0.74	1.24	3.05
50th percentile	2.07	5.66	5.87	2.02	4.59	4.54
75th percentile	2.30	7.57	8.41	2.42	6.38	7.44
Mean	2.30	6.44	6.13	1.77	4.76	5.31

**Table 4:** Results of multivariate analysis for the head scans performed using MDCT_64_.

	Predictor	*B*	β	*t* ^a^	VIF ^b^
Head CTA (*R*^2^ = 0.9618)	Body size	−0.0197	−0.0276	−1.82	1.08
	kVp^2^	0.0031	0.6285	41.66	1.07
	mAs	0.1484	0.6552	44.24	1.03
	1/pitch	29.9818	0.4221	28.45	1.04
Head scan excluding CTA (*R*^2^ = 0.9068)	Body size	−0.0074	−0.0060	−1.35	1.04
	kVp^2^	0.0047	0.4438	83.54	1.45
	mAs	0.1667	0.8993	176.61	1.33
	1/pitch	31.9752	0.5067	91.81	1.57

^a^ A predictor is considered to be statistically significant if |*t*| > 2. ^b^ A maximum variance inflation factor (VIF) value in excess of 10 is taken as an indication that multicollinearity may be unduly influencing the least square estimates.

**Table 5:** Results of multivariate analysis for the chest scans performed using MDCT_64_.

	Predictor	*B*	β	*t* ^a^	VIF ^b^
Chest CTA (*R*^2^ = 0.8218)	Body size	0.0085	0.0808	3.56	1.26
	kVp^2^	0.0010	0.3303	15.28	1.14
	mAs	0.0568	0.8709	37.51	1.31
	1/pitch	11.5789	0.2922	13.81	1.09
Chest scan excluding CTA (*R*^2^ = 0.8223)	Body size	0.0074	0.0651	7.21	1.48
	kVp^2^	0.0010	0.3189	39.41	1.12
	mAs	0.0609	0.8239	89.53	1.54
	1/pitch	9.8958	0.3064	41.15	1.01

^a^ A predictor is considered to be statistically significant if |*t*| > 2. ^b^ A maximum VIF value in excess of 10 is taken as an indication that multicollinearity may be unduly influencing the least square estimates.

**Table 6:** Results of multivariate analysis for the abdominal scans performed using MDCT_64_.

	Predictor	*B*	β	*t* ^a^	VIF ^b^
Abdominal CTA (*R*^2^ = 0.9146)	Body size	0.0125	0.1236	12.25	2.97
	kVp^2^	0.0010	0.7781	82.72	2.58
	mAs	0.0566	0.7250	68.62	3.26
	1/pitch	5.0738	0.4329	45.80	2.61
Abdominal scan excluding CTA (*R*^2^ = 0.9096)	Body size	0.0075	0.0666	13.12	1.63
	kVp^2^	0.0011	0.7576	147.97	1.66
	mAs	0.0628	0.9698	165.19	2.18
	1/pitch	6.5695	0.6559	130.98	1.59

^a^ A predictor is considered to be statistically significant if |*t*| > 2. ^b^ A maximum VIF value in excess of 10 is taken as an indication that multicollinearity may be unduly influencing the least square estimates.

**Table 7:** Results of multivariate analysis for the head scans performed using MDCT_H_ (*R*^2^ = 0.8854).

Predictor	B	β	t ^a^	VIF ^b^
Body size	−0.0621	0.0758	−1.95	1.02
kVp^2^	--- ^c^	---	---	---
mAs	0.1777	0.6700	48.98	1.00
1/pitch	44.0395	0.6851	46.53	1.03

^a^ A predictor is considered to be statistically significant if |*t*| > 2. ^b^ A maximum VIF value in excess of 10 is taken as an indication that multicollinearity may be unduly influencing the least square estimates. ^c^ kVp^2^ was not included in multivariate analysis.

**Table 8:** Results of multivariate analysis for the chest scans performed using MDCT_H_.

	Predictor	*B*	β	*t* ^a^	VIF ^b^
Chest CTA (*R*^2^ = 0.9296)	Body size	0.0086	0.0214	2.01	1.12
	kVp^2^	0.0042	0.6232	40.62	2.10
	mAs	0.1444	0.6945	31.17	4.43
	1/pitch	7.0585	0.6492	27.85	4.84
Chest scan excluding CTA (*R*^2^ = 0.7903)	Body size	0.0143	0.1456	20.37	1.06
	kVp^2^	0.0004	0.0817	11.50	1.05
	mAs	0.0481	0.7914	105.02	1.18
	1/pitch	5.7302	0.6219	82.83	1.17

^a^ A predictor is considered to be statistically significant if |*t*| > 2. ^b^ A maximum VIF value in excess of 10 is taken as an indication that multicollinearity may be unduly influencing the least square estimates.

**Table 9:** Results of multivariate analysis for the abdominal scans performed using MDCT_H_.

	Predictor	*B*	β	*t* ^a^	VIF ^b^
Abdominal CTA (*R*^2^ = 0.9167)	Body size	0.0141	0.1817	13.84	4.49
	kVp^2^	0.0003	0.0990	10.99	2.11
	mAs	0.0932	0.7816	73.91	2.91
	1/pitch	0.2939	0.0206	3.27	1.03
Abdominal scan excluding CTA (*R*^2^ = 0.7879)	Body size	0.0393	0.3626	35.22	1.30
	kVp^2^	0.0008	0.0842	9.33	1.00
	mAs	0.0475	0.5910	56.92	1.33
	1/pitch	3.8132	0.3130	33.71	1.06

^a^ A predictor is considered to be statistically significant if |*t*| > 2. ^b^ A maximum VIF value in excess of 10 is taken as an indication that multicollinearity may be unduly influencing the least square estimates.

## References

[1] Aroua A., Samara E.T., Bochud F.O., Meuli R., Verdun F.R. (2013). Exposure of the Swiss population to computed tomography. BMC Med. Imaging.

[2] Kalender W.A. (2014). Dose in x-ray computed tomography. Phys. Med. Biol..

[3] Mettler F.A., Bhargavan M., Faulkner K., Gilley D.B., Gray J.E., Ibbott G.S., Lipoti J.A., Mahesh M., McCrohan J.L., Stabin M.G. (2009). Radiologic and nuclear medicine studies in the United States and worldwide: Frequency, radiation dose, and comparison with other radiation sources—1950–2007. Radiology.

[4] Goldman L.W. (2007). Principles of CT and CT technology. J. Nucl. Med. Technol..

[5] Goldman L.W. (2008). Principles of CT: Multislice CT. J. Nucl. Med. Technol..

[6] Tonkopi E., Duffy S., Abdolell M., Manos D. (2017). Diagnostic Reference Levels and Monitoring Practice Can Help Reduce Patient Dose From CT Examinations. Am. J. Roentgenol..

[7] Smith-Bindman R., Moghadassi M., Wilson N., Nelson T.R., Boone J.M., Cagnon C.H., Gould R., Hall D.J., Krishnam M., Lamba R. (2015). Radiation Doses in Consecutive CT Examinations from Five University of California Medical Centers. Radiology.

[8] (1991). 1990 Recommendations of the International Commission on Radiological Protection. Ann. ICRP.

[9] (2001). Diagnostic reference levels in medical imaging: Review and additional advice. Ann. ICRP.

[10] Roch P., Aubert B. (2013). French diagnostic reference levels in diagnostic radiology, computed tomography and nuclear medicine: 2004-2008 review. Radiat. Prot. Dosimetry..

[11] Simantirakis G., Hourdakis C.J., Economides S., Kaisas I., Kalathaki M., Koukorava C., Manousaridis G., Pafilis C., Tritakis P., Vogiatzi S. (2015). Diagnostic reference levels and patient doses in computed tomography examinations in Greece. Radiat. Prot. Dosimetry..

[12] Foley S.J., McEntee M.F., Rainford L.A. (2012). Establishment of CT diagnostic reference levels in Ireland. Br. J. Radiol..

[13] Palorini F., Origgi D., Granata C., Matranga D., Salerno S. (2014). Adult exposures from MDCT including multiphase studies: First Italian nationwide survey. Eur. Radiol..

[14] Kim M.C., Han D.K., Nam Y.C., Kim Y.M., Yoon J. (2015). Patient dose for computed tomography examination: Dose reference levels and effective doses based on a national survey of 2013 in Korea. Radiat. Prot. Dosimetry.

[15] Santos J., Foley S., Paulo G., McEntee M.F., Rainford L. (2014). The establishment of computed tomography diagnostic reference levels in Portugal. Radiat. Prot. Dosimetry.

[16] Treier R., Aroua A., Verdun F.R., Samara E., Stuessi A., Trueb P.R. (2010). Patient doses in CT examinations in Switzerland: Implementation of national diagnostic reference levels. Radiat. Prot. Dosimetry.

[17] Thakur Y., Bjarnason T.A., Baxter P., Griffith M., Eaton K. (2016). Radiation Dose Survey for Common Computed Tomography Exams: 2013 British Columbia Results. Can. Assoc. Radiol. J..

[18] Matsunaga Y., Kawaguchi A., Kobayashi K., Kinomura Y., Kobayashi M., Asada Y., Minami K., Suzuki S., Chida K. (2015). Survey of volume CT dose index in Japan in 2014. Br. J. Radiol..

[19] Charnock P., Dunn A.F., Moores B.M., Murphy J., Wilde R. (2015). Establishment of a comprehensive set of regional DRLs for CT by means of electronic X-ray examination records. Radiat. Prot. Dosimetry.

[20] (2007). The 2007 Recommendations of the International Commission on Radiological Protection. ICRP publication 103. Ann. ICRP.

[21] MacDougall R.D., Kleinman P.L., Callahan M.J. (2016). Size-based protocol optimization using automatic tube current modulation and automatic kV selection in computed tomography. J. Appl. Clin. Med. Phys..

[22] Martin C.J., Sookpeng S. (2016). Setting up computed tomography automatic tube current modulation systems. J. Radiol. Prot..

[23] Yu L., Bruesewitz M.R., Thomas K.B., Fletcher J.G., Kofler J.M., McCollough C.H. (2011). Optimal tube potential for radiation dose reduction in pediatric CT: Principles, clinical implementations, and pitfalls. Radiographics.

[24] Zacharias C., Alessio A.M., Otto R.K., Iyer R.S., Philips G.S., Swanson J.O., Thapa M.M. (2013). Pediatric CT: Strategies to lower radiation dose. Am. J. Roentgenol..

[25] You S.K., Choi Y.H., Cheon J.E., Kim W.S., Kim I.O., Lee S.M., Cho H.H. (2019). Effect of low tube voltage and low iodine concentration abdominal CT on image quality and radiation dose in children: Preliminary study. Abdom. Radiol..

[26] Geyer L.L., Schoepf U.J., Meinel F.G., Nance J.W., Bastarrika G., Leipsic J.A., Paul N.S., Rengo M., Laghi A., De Cecco C.N. (2015). State of the Art: Iterative CT Reconstruction Techniques. Radiology.

[27] Klink T., Obmann V., Heverhagen J., Stork A., Adam G., Begemann P. (2014). Reducing CT radiation dose with iterative reconstruction algorithms: The influence of scan and reconstruction parameters on image quality and CTDIvol. Eur. J. Radiol..

[28] Padole A., Ali Khawaja R.D., Kalra M.K., Singh S. (2015). CT radiation dose and iterative reconstruction techniques. Am. J. Roentgenol..

[29] Shan H., Padole A., Homayounieh F., Kruger U., Khera R.D., Nitiwarangkul C., Kalra M.K., Wang G. (2019). Competitive performance of a modularized deep neural network compared to commercial algorithms for low-dose CT image reconstruction. Nat. Mach. Intell..

[30] Shan H., Zhang Y., Yang Q., Kruger U., Kalra M.K., Sun L., Cong W., Wang G. (2018). 3-D Convolutional Encoder-Decoder Network for Low-Dose CT via Transfer Learning From a 2-D Trained Network. IEEE Trans. Med. Imaging.

[31] Wolterink J.M., Leiner T., Viergever M.A., Išgum I. (2017). Generative Adversarial Networks for Noise Reduction in Low-Dose CT. IEEE Trans. Med. Imaging.

[32] Halliburton S., Arbab-Zadeh A., Dey D., Einstein A.J., Gentry R., George R.T., Gerber T., Mahesh M., Weigold W.G. (2012). State-of-the-art in CT hardware and scan modes for cardiovascular CT. J. Cardiovasc. Comput. Tomogr..

[33] Lewis M.A., Pascoal A., Keevil S.F., Lewis C.A. (2016). Selecting a CT scanner for cardiac imaging: The heart of the matter. Br. J. Radiol..

[34] Dey D., Slomka P.J., Berman D.S. (2014). Achieving very-low-dose radiation exposure in cardiac computed tomography, single-photon emission computed tomography, and positron emission tomography. Circ. Cardiovasc. Imaging.

